# Case Report: Neonatal Unexplained HUS Treated With Complement Inhibitor Eculizumab

**DOI:** 10.3389/fped.2020.579607

**Published:** 2021-02-18

**Authors:** Dorottya Kelen, Benedetta Chiodini, Valérie Godart, Brigitte Adams, Patrick Stordeur, Khalid Ismaili

**Affiliations:** ^1^Neonatal Department, Hôpital Erasme, Université Libre de Bruxelles, Brussels, Belgium; ^2^Department of Pediatric Nephrology, Hôpital Universitaire des Enfants Reine Fabiola, Université Libre de Bruxelles, Brussels, Belgium; ^3^Belgian National Reference Centre for the Complement System, Laboratory of Immunology, LHUB-ULB, Université Libre de Bruxelles, Brussels, Belgium

**Keywords:** hemolytic uremic syndrome, perinatal asphyxia, eculizumab, neonatal, case report

## Abstract

**Background:** Hemolytic uremic syndrome (HUS) is rare in neonates. It is probably an under-recognized condition in the early postnatal period as it presents similarly to the most common perinatal asphyxia and to differentiate the two conditions is challenging. We describe the clinical presentation of a potential new subtype of neonatal HUS triggered by hypoxic-ischemic event. Our patient was successfully treated by a single dose of Eculizumab as early as at 9 days of life.

**Case Report:** A 35-weeks infant was born with low hemoglobin and subsequently developed respiratory distress, hypotension, and acidosis. Blood transfusion was administered, acidosis corrected, neurological examination remained reassuring. Few hours later he developed renal failure, macroscopic hematuria, hemobilia, thrombocytopenia and coagulopathy refractory to platelet and fresh frozen plasma transfusions. No infection was found. Haptoglobin was non-measurable, and schistocytes present, complement factors C3, C4 and B were low, FBb increased. HUS was suspected. A single dose of Eculizumab™ was administered on day 9 of life. No genetic mutation of atypical HUS was found. He was discharged with improving renal function and developing cholestasis.

**Conclusion:** In neonates with hemolytic anemia, thrombocytopenia, hematuria and renal failure, HUS should be suspected. In neonatal HUS Eculizumab should be considered as first-line therapy and discontinuation can be considered if no genetic mutation is found and clinical condition improves. In very young patients, cholestasis could appear as potential side effect of Eculizumab™.

## Introduction

Hemolytic uremic syndrome (HUS) is rare in neonates. It has been observed in von Willebrand factor-cleaving protease (ADAMTS13) deficiency, inborn errors of cobalamin absorption and metabolism, and alternative pathway complement system deficiencies (1). Exceptionally it has been related to *E. coli* shiga toxins (STEC) (2).

However, neonatal HUS could also be triggered by a hypoxic-ischemic event and this is probably an under-recognized condition as in the early postnatal period HUS presents similarly to the more common perinatal asphyxia (PA). To differentiate the two conditions is challenging. We describe the clinical presentation of a potential new subtype of neonatal HUS.

Our patient was successfully treated by a single dose of Eculizumab as early as at 9 days of life.

## Case Discussion

A 35-week 4 days gestational age male infant was born by cesarean section for placenta praevia, with birth weight of 3,080 g. Maternal Kleinhauer and coombs test were negative, and maternal renal function was normal (creatinine 0.4 mg/dL). The newborn had good Apgar scores (9/9/9), and normal cord pH, but low Hb (11.6 g/dL). He was pale and developed hypotension with a blood pressure (BP) of 29/16 mmHg. Non-invasive respiratory support was started as he also developed respiratory distress (peripheral oxygen saturation 86%, heart rate 150–170/min). At 1 h of life, his blood gas showed anemia and mixed acidosis (pH 6.9, pCO_2_ 62 mmHg, BE −19 mmol/L, lactate 12 mmol/L, Hb 7.8 g/dL). However, neurological criteria for PA *j*ustifying therapeutic hypothermia were not met (3). Following administration of cross-matched red blood cell transfusion, his neurological examination was still reassuring, and his breathing, blood pressure and gas analysis normalized (peripheral oxygen saturation >95% in air, BP 53/29 mmHg, lactate 1.9 mmol/l). At 30 h of life he developed macroscopic hematuria, oliguria, hypertension, severe renal failure and hyperkalemia (K^+^ 9.5 mmol/L, urea 71.6 mg/dL, creatinine 3 mg/dL). Liver enzymes were elevated (AST 2013 U/L, ALT 625 UI/L), serum LDH increased (9,920 U/L), while CK levels were normal (820 U/L). He also showed signs of anemia, thrombocytopenia and coagulopathy (Hb 11.5 g/dL, Plt 23.000/mm^3^, INR 2.76, fibrinogen 82 mg/dL, D-dimer >35,000 ng/ml FEU). Antibiotics were started in view of the clinical deterioration, although no infection was found. Renal ultrasound showed bilateral cortical hyperechogenicity with no signs of renal thrombosis.

On day 3, cranial ultrasound, amplitude-integrated electroencephalogram, and neurological clinical examination were still reassuring. The infant received a second blood transfusion, fresh frozen plasma (FFP) and platelet transfusion. Then a second platelet transfusion and FFP for 6 consecutive days at a dose of 15 ml/kg were administered, with no improvement in coagulation or platelet count, and further deterioration of the renal function. His BP was still elevated with a maximum measured of 90/58 mmHg. In addition to hematuria, hemobilia was observed without hyperbilirubinemia.

As there was no clear etiology of the multi-organ failure (MOF) and disseminated intravascular coagulopathy (DIC), and neurological criteria for asphyxia were not met, investigations toward HUS have been initiated.

Haptoglobin was non-measurable, his peripheral blood smear test showed signs of hemolysis and schistocytes count was at about 10%. Cobalamin and ADAMTS 13 were within the normal range for age, and ANCA, ANA and anti-FH antibodies were not detected. Concentration of the following components of the complement system (measured 48 h after FFP administration) appeared decreased: C3 27 mg/dL (normal range: 80–164 mg/dL), C4 5 mg/dL (normal range: 10–46 mg/dL), Factor B 6 mg/dL (normal range: 11–22 mg/dL), Factor I 3.9 mg/dL (normal range: 4.0–10.7 mg/dL), and Factor H 34 mg/dL (normal range: 37–73 mg/dL). CD46 (MCP) expression was normal. Even though these tests were carried out after FFP administration and the normal values for adults are not adapted to neonates, the observed results suggested an alteration of the alternative pathway, confirmed by a significant increase of the FBb concentration [FBb 0.68 mg/dL (<0.15)]. Genetic test results for atypical HUS showed eventually no mutations in CFH, CFI, CFB, C3, CD46 (MCP), Thrombomodulin, CFHR1-R3 deletion, Recombinant CFH/CFHR1 hybrid, and DGKE genes.

**Figure 1:**
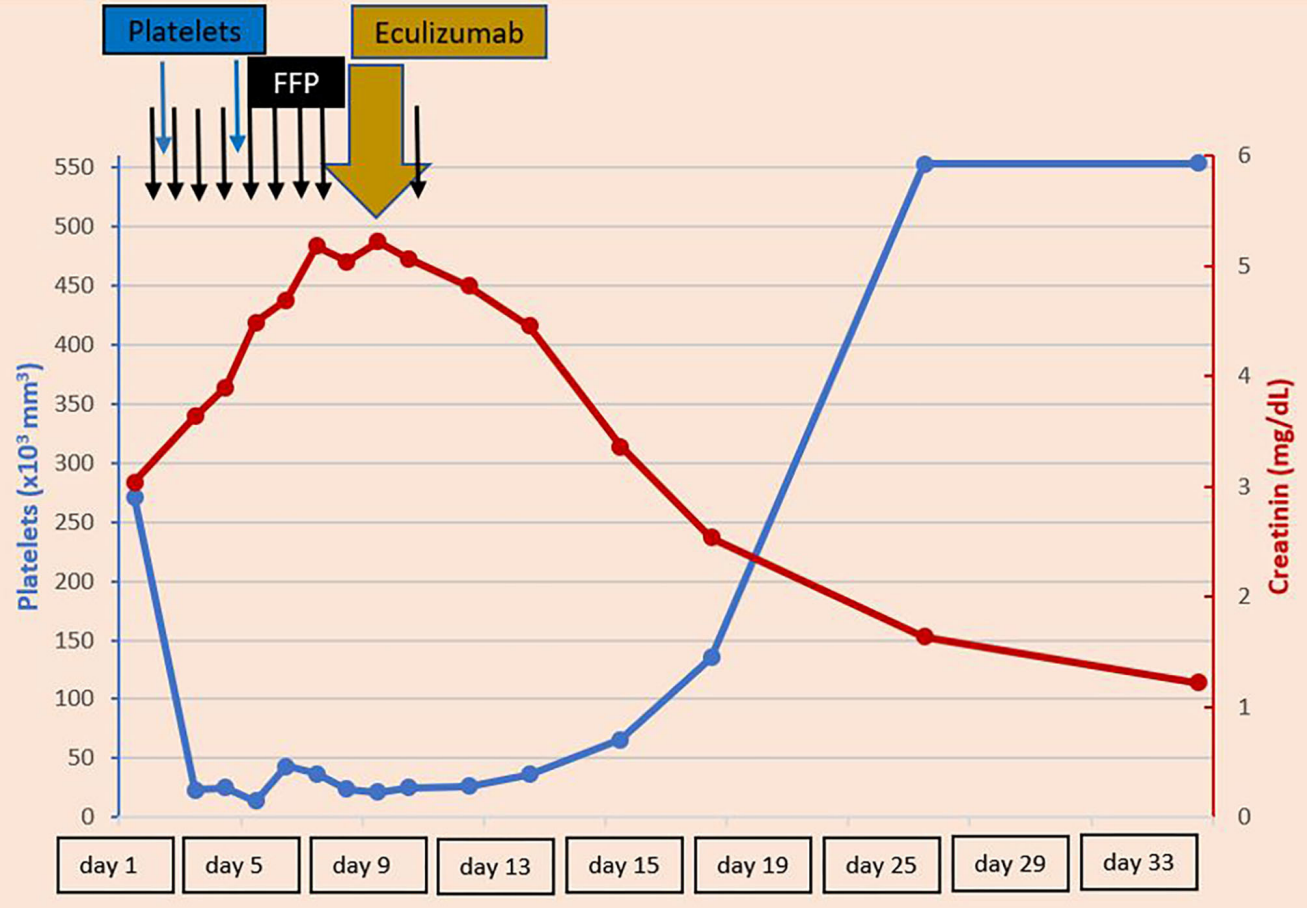
Platelets and creatinine levels during the first 33 days of patient's life—Refractory thrombocytopenia and severe renal failure developed on day 2 of life. Improvements have been observed after administration of Eculizumab on day 9 of life. FFP, Fresh frozen plasma.

In this clinical situation, on day 9 of life, 300 mg of Eculizumab™ were administered. Antibiotic prophylaxis was given, and vaccination completed as soon as 8 weeks of age. Kidney function started to improve 3 days after the administration of Eculizumab, while coagulation and platelet count improved between days 5 and 14 (Figure 1). Gamma-glutamyl transferase (GGT) levels, which were initially normal, started to increase.

The baby was discharged on day 29 of life with improving renal function (urea 78.4 mg/dL, creatinine 1.64 mg/dL), and developing cholestasis. At 3 months of life, he underwent endoscopy resolving his cholestasis, his BP at that time was 115/55 mmHg.

At 10 months of life, he showed an improved renal function (urea 30 mg/dL, creatinine 0.53 mg/dL), and normalized values of hematology (Hb 10.2 g/dL, Plt 449 × 10^3^ /μL), liver enzymes (GOT 35 UI/L, GPT 26 UI/L, Serum bilirubin < 0.2 mg/dL, GGT 24 UI/L), and complement components (C3 0.82 g/L, C4 0.15 g/L, FBb 0.1 mg/dL, Haptoglobin 130 mg/dL LDH 269 UI/L, schistocytes 4/1,000 GR).

## Discussion

In neonates presenting with acute kidney injury (AKI), MOF and coagulopathy, it is often difficult to distinguish between DIC, PA, and neonatal HUS. Few elements can help in the differential diagnosis.

Usually PA manifests with neurological signs directly after birth, while symptoms of MOF and DIC develop subsequently (1). In HUS and sepsis neurological signs develop further to MOF and DIC. Gross hematuria is often an early sign in neonatal HUS, as well as severe thrombocytopenia (4, 5). Schistocytes are classically present in HUS and are generally low or not found in the other conditions (2) (Table 1).

**Table 1:** Elements to help in the differential diagnosis between perinatal asphyxia (PA), disseminated intravascular coagulation (DIC), and neonatal hemolytic uremic syndrome (HUS).

**Characteristics**	**PA**	**DIC**	**Neonatal HUS**
Haemolytic anemia	–	–/+	+++
Increased level of LDH/CK	++/+++	+/–	+++/–
Thrombocytopenia	–	++	+++
Schistocytes	–	+/–	+++
AKI	+/–	–	+++
Haematuria	–	+/–	+++
Coagulopathy	+/–	+++	+++
Early neurological signs	+++	–/+	–/+
Alteration of complement system	–	+/–	+++
Blood pressure	↓/↑	↓	↑↑

Our patient presented with anemia, thrombocytopenia and AKI without neurological signs, and soon developed gross hematuria with extremely high D-dimers. Altogether it was not a typical picture of PA.

A hypoxic-ischemic perinatal event, in our case hypotension due to possible acute blood loss from the placenta previa, might have caused endothelial cell damage leading to a vicious circle of consumption of platelets and plasma factors which then triggered HUS. The endothelial cell injury caused by ischemia could have been the starting point of the micro-angiopathic cascade. Biran et al. have hypothesized a similar mechanism on three neonates who developed renal failure with biological features compatible with HUS following a hypoxic-ischemic perinatal event (1). Our diagnosis was based on extremely high FBb levels demonstrating complement activation (6), although interpretation of complement results was compromised by previous FFP administration and reference intervals adopted to adults but not to neonates. Concentration of most of the complement factors is indeed lower in neonates compared to children and adults, and even lower in preterm neonates (7). This is especially the case of FH, FI and FB (8).

Absence of well-defined reference intervals for neonates is not the only problem to face in complement testing. The other main drawback is the absence of standardization of the techniques used to measure the parameters of the complement system (7).

Since 2011, Eculizumab, a recombinant human monoclonal anti-C5 antibody is approved by FDA for the treatment of atypical HUS (9).

An international group of experts suggests Eculizumab as first-line treatment for children with clinical diagnosis of atypical HUS even when no genetic mutations are found (10).

Eculizumab has been successfully used in a few cases of neonatal HUS, most of them presenting a factor H mutation (4, 5, 11, 12). In all cases Eculizumab has been administered long term, usually on a regime of 300 mg every 3 week. Our patient received 300 mg Eculizumab on day 9 of life. To our best knowledge, he is the youngest newborn with HUS successfully treated with the anti-C5 monoclonal antibody. In view of his improving clinical condition, and the absence of a consensus about the duration of the therapy without underlying genetic condition, we stopped the treatment after a unique dose of Eculizumab (10). The risks of prolonged Eculizumab therapy are not negligible and include immunosuppression, need for prophylactic antibiotics, and high financial costs.

The appearance of cholestasis, observed few days after Eculizumab administration is a potential side effect already described in young STEC-HUS cases (13). It is still uncertain if the delayed cholestasis is a side effect of Eculizumab itself or an unusual complication of the HUS disease. However, Mauras et al. suggest that this risk should be kept in mind in patients receiving Eculizumab at a very young age (13).

## Conclusion

In neonatal cases with history of a hypoxic- ischemic event, presenting with hemolytic anemia, thrombocytopenia, gross hematuria and AKI, HUS should be suspected. In this scenario, genetic tests toward atypical HUS are required, and complement factors should be analyzed before FFP administration. In case of neonatal HUS Eculizumab should be considered as first-line therapy and FFP administered until the anti-C5 antibody is available. Liver enzymes should be monitored following the administration of Eculizumab. In HUS triggered by perinatal hypoxic- ischemic events when no genetic mutations are found, stopping Eculizumab therapy can be considered in case of improving clinical conditions.

## Data Availability Statement

The original contributions presented in the study are included in the article/supplementary material, further inquiries can be directed to the corresponding author/s.

## Ethics Statement

Ethical review and approval was not required for the study on human participants in accordance with the local legislation and institutional requirements. Written informed consent to participate in this study was provided by the participants' legal guardian/next of kin. Written informed consent was obtained from the minor(s)' legal guardian/next of kin for the publication of any potentially identifiable images or data included in this article.

## Author Contributions

DK: physician in charge of the clinical care of the case, drafting the manuscript, revising the manuscript, final approval of the version to be published. BC: drafting the manuscript, revising the manuscript, final approval of the version to be published. VG: drafting the case report in the manuscript, final approval of the version to be published. BA: revising the manuscript, final approval of the version to be published. PS: complement factors measurements, analysis and interpretation of data, revising the manuscript, final approval of the version to be published. KI: specialist contribution to the clinical treatment, revising the manuscript, final approval of the version to be published. All authors contributed to the article and approved the submitted version.

## Conflict of Interest

PS has received fees from Alexion Pharmaceuticals through his employer. The remaining authors declare that the research was conducted in the absence of any commercial or financial relationships that could be construed as a potential conflict of interest.

## Abbreviations

HUS: Hemolytic uremic syndrome
FFP: Fresh frozen plasma
DIC: Disseminated intravascular coagulopathy
MOF: multiorgan failure
PA: Perinatal asphyxia
AKI: acute kidney injury.
